# Cross-over data supporting long-term antibiotic treatment in patients with painful lower urinary tract symptoms, pyuria and negative urinalysis

**DOI:** 10.1007/s00192-018-3846-5

**Published:** 2018-12-18

**Authors:** Sheela Swamy, Anthony S. Kupelian, Rajvinder Khasriya, Dhanuson Dharmasena, Hristina Toteva, Tara Dehpour, Linda Collins, Jennifer L. Rohn, James Malone-Lee

**Affiliations:** 1 Centre for Nephrology, Division of Medicine, University College London, London, UK; 2 University College London Hospitals NHS Trust, London, UK; 3 Whittington Hospitals NHS Foundation Trust, London, UK

**Keywords:** Recurrent UTI, Chronic UTI, Bladder pain syndrome, Interstitial cystitis, UTI guidelines, Painful lower urinary tract symptoms

## Abstract

**Purpose:**

To measure the effects of an unplanned, sudden cessation of treatment in an unselected group of patients with chronic painful LUTS managed with protracted antimicrobial treatment and to report these observational data collected from a cross-over process.

**Materials and methods:**

The imposition of a guideline resulted in the immediate cessation of antibiotic treatment in a cohort of patients with chronic painful LUTS and microscopic pyuria. Patients were assessed before treatment withdrawal, whilst off treatment, and following reinstatement. Outcome measures included a validated symptom score, microscopic enumeration of urinary white cells and uroepithelial cells, and routine urine culture.

**Results:**

These patients had reported treatment-resistant, painful LUTS for a mean of 6.5 years before treatment at this centre. Treatment was stopped in 221 patients (female = 210; male = 11; mean age = 56 years; SD = 17.81). Sixty-six per cent of women were post-menopausal. After unplanned treatment cessation, 199 patients (90%; female = 188; male = 9) reported deterioration. Eleven patients required hospital care in association with disease recurrence, including acute urinary tract infection (UTI) and urosepsis. Symptom scores increased after cessation and recovered on reinitiating treatment (F = 33; df = 2; *p* < 0.001). Urinary leucocyte (F = 3.7; df = 2; *p* = 0.026) and urothelial cells counts mirrored symptomatic changes (F = 6.0; df = 2; *p* = 0.003). Routine urine culture results did not reflect changes in disease status.

**Conclusion:**

These data support the hypothesis that treating painful LUTS associated with pyuria with long-term antimicrobial courses, despite negative urine culture, is effective. The microscopy of fresh unspun, unstained urine to count white cells and epithelial cells offers a valid method of monitoring disease. An unplanned cessation of antibiotic therapy produced a resurgence of symptoms and lower urinary tract inflammation in patients with chronic LUTS, supporting an infective aetiology below the level of routine detection.

## Introduction

Recalcitrant chronic bladder pain and symptoms of recurrent cystitis in patients with negative urinalysis present a worrying management problem. The treatment of painful lower urinary tract symptoms (LUTS) is a significant challenge, and there are few quality data to guide clinicians. The evidence for oral or intravesical therapies for painful bladder symptoms is poor [1]. Cystodistension and urethral dilation are also deployed without evidence to support their utility [2–4]. Patients with recurrent, acute symptoms of cystitis and negative urinalysis are often exposed to multiple, short courses of antibiotics in primary care without evidence of benefit [5].

While there are published guidelines for managing acute and recurrent urinary tract infection (UTI) [6], there are none for patients who may be suffering a chronic form of the disease. Recently, we published data describing our experience treating women with chronic painful LUTS for a mean duration of 6 years prior to presentation to our clinic. These patients had been exposed to conventional therapies without a resolution of symptoms. Data were gathered from 624 patients over 10 years. Given microscopic pyuria and symptoms, we presumed an infectious cause even in the face of negative dipstick urinalysis and urine cultures, as these tests lack sensitivity and fail to detect infective organisms in bona fide disease [7, 8]. This previous report described the evolution of a simple management regimen using first-generation, narrow-spectrum, urinary antibiotics combined with methenamine hippurate, guided by symptom palliation and the resolution of pyuria [9]. The study demonstrated that treatment was associated with symptomatic improvement and the resolution of biomarkers of infection. There were few treatment-emergent adverse events (AEs) and no significant increase in antibiotic-resistant isolates. These data are being employed to develop a randomised trial (RCT).

This novel approach requires clinic-based fresh urine microscopy to quantify pyuria, full doses of urinary antibiotic, protracted treatment periods and careful safety monitoring [9]. Although some patients (20%) could be discharged after only 6 months, we found that it took an average of 383 days [95% confidence interval (CI) = 337–428] to achieve symptom resolution (mean reduction in validated symptom score = 70%) without the need for further antibiotic treatment. A reduction in symptoms and pyuria associated with antibiotic treatment, followed by deterioration in both measures on early treatment cessation, provided additional cogent observational evidence of efficacy.

Despite positive preliminary data, this therapeutic approach contravenes guidelines for treating acute urinary infection and recognised treatment regimens for recurrent UTI [10]. It also defies antibiotic stewardship policies [11, 12]. The evolution of this treatment method has been supported by a long-running translational research programme and basic scientific activity at University College London. As such, the clinical approach has been founded on peer-reviewed preclinical and clinical research in addition to the systematic collection of efficacy and safety data through surveillance. Nevertheless, the nature of the treatment generated understandable concern amongst medical managers within the host NHS trust and primary care commissioners.

In September 2015 a patient being managed with nitrofurantoin, having failed numerous attempts to withdraw this agent over 30 months, developed a rare, acute eosinophilic pneumonitis, which is reported to occur only during the first 6 months of treatment [13]. Nitrofurantoin was stopped, and the occurrence was reported to our trust, in line with local safety monitoring procedures. This one serious adverse event (SAE) occurred amongst a cohort of 624 patients during 273,762 treatment days. There were 475 total AEs during this period, managed with medication withdrawal or modification as outpatients.

In response to this SAE, the trust medical management imposed prescribing restrictions limiting treatment to short-course antibiotic therapy recommended by guidelines for acute UTI. This imposition effectively suspended the service, and treatment was stopped in 221 patients on long-term regimens. Although the service reopened 5 weeks later, this sudden suspension tested the hypothesis, supported by previous data, that premature cessation of long-term antibiotic therapy results in disease regression. This article reports the outcomes that ensued from this event.

## Methods

Our diagnosis of UTI rests on symptoms, signs, and microscopic pyuria. Without the latter we do not initiate antibiotic treatment. We combine methenamine hippurate, a bactericidal urinary antiseptic, with a full-dose, first-generation, narrow-spectrum urinary antibiotic. Antibiotic selection is based on symptomatic response and a reduction in pyuria along with drug tolerance. Cefalexin is favoured as first-line therapy because previous outcome data showed this first-generation cephalosporin to evince the least side effect record of all of the agents that we had used. It is known to have the lowest *Clostridium difficile* incidence of the antibiotics [14]. Trimethoprim and nitrofurantoin were second- and third-choice agents. We continue treatment until symptom control is optimal and pyuria has cleared before testing treatment withdrawal; we restart the treatment if relapse occurs. Usually, more than one cycle is required to achieve lasting symptom resolution off treatment [9].

The clinical service was suspended for 5 weeks from 21 October 2015. When these restrictions were lifted, we contacted patients who had stopped treatment. We identified those who reported symptom deterioration and wherever possible assessed them at the centre as a priority. We measured their symptoms, using a validated measure [15], and urine samples were examined immediately by microscopy using a haemocytometer to quantify leucocytes and shed epithelial cells. Previous work using an antibody against the specific urothelial marker protein uroplakin-3 has shown that the majority of epithelial cells present in the urine specimens of these patients originate from the bladder, they are not contaminants from the vulva or vagina [16]. In the majority of cases, disease recurrence, indicated by worsening symptoms and pyuria, motivated reintroduction of treatment. The clinic suspension permitted the collection of data before treatment cessation, whilst off treatment following cessation and after treatment was restarted.

The following variables were collected: 24-h frequency, 24-h incontinence episodes, lower urinary tract pain, urinary urgency, voiding symptoms and stress urinary incontinence [15]. If attending the centre, urinalysis included urinary leucocyte and epithelial cell counts, quantified from fresh urinary microscopy, and routine urine culture. These data were reported at three time points: (1) whilst on treatment prior to the closure; (2) whilst off treatment after the closure; (3) after recommencing treatment. Data reported after restarting treatment were captured from the last consultation within 12 months of the cessation. Some of the symptomatic data were gathered by telephone consultation only, using the measures outlined above. Telephone reviews did not permit urinary biomarker data to be collected at all consultations.

The East Central London Regional Ethics Committee (REC1) (Ref: 11/H0721/7) provided ethical approval for data collection.

## Statistics

We used the IBM SPSS version 25 (IBM, New York) for analyses. The data were tested for normality using Q-Q plots. A close linear relationship between the measured variables and the theoretical Z-scores existed and so the data were suitable for parametric analysis. We analysed the differences over the three assessment points using a repeated measures ANOVA. Mauchly’s test was used and common variance was not violated.

## Results

The unplanned cessation of treatment occurred in 221 patients (female = 210; male = 11) of the 1035 active patients at the service, with a mean age of 56 years (range = 19–92; SD = 17.81). Sixty-six per cent of the women were post-menopausal. They had experienced treatment-resistant, painful lower urinary tract symptoms (LUTS) for a mean of 6 years (SD = 7) prior to presentation at this centre. They had attended the centre for an average of seven clinic visits (SD = 6) over a mean of 1.7 years (SD = 2), and all 1035 active patients were being treated with antibiotics with regular trials of cessation. One hundred and ninety-nine patients (90%; female = 188; male = 9) reported deterioration in their symptoms after stopping treatment. Thus, 21 did not report deterioration (10%; female = 19; male = 2). We collected data on 192 (97%) of those who deteriorated. The other seven were unavailable to provide the minimum data set of symptom measures at three assessments. Eight hundred and fourteen patients on our books did not have their treatment interfered with during the 4-week clinic suspension as they were either not due an appointment during this period or some managed to renew their prescription from the GP or private gynaecologist.

Patients had been assessed an average of 58 days (SD = 49) before clinic closure. The service was closed for 5 weeks and the patients were first reviewed an average of 68 days (SD = 38) after the closure. Their last review, within a year of the suspension, was a mean of 284 days after clinic closure (SD =76).

**Fig. 1:**
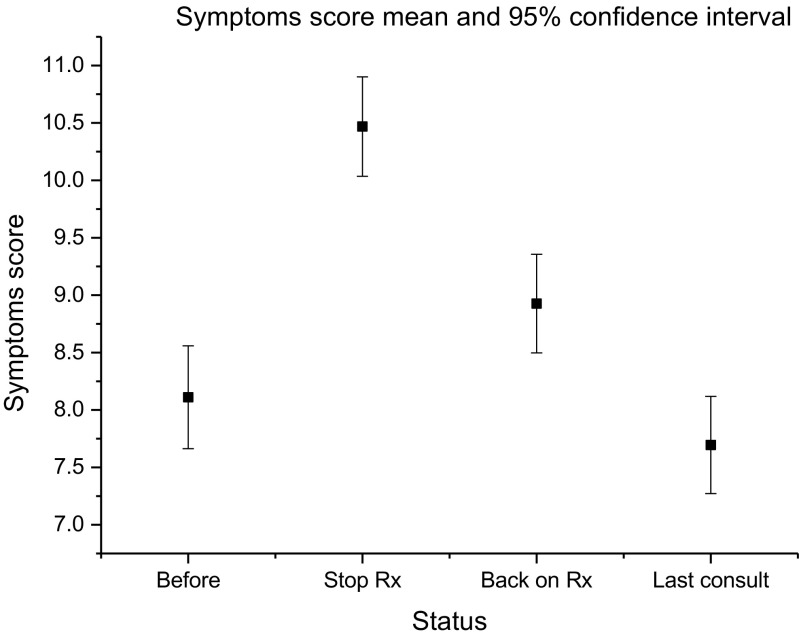
Mean total symptom score before cessation, when off treatment and following reintroduction

The results of the statistical analysis are reported in Table 1. There was an increase in symptoms after cessation followed by recovery in the wake of treatment reinstatement (F = 33; df = 2; *p* < 0.001). Figure 1 illustrates the symptom response, including data from the last patient review (mean = 353 days; SD = 70; female = 21; df = 3; *p* < 0.001). Symptom scores increased after antibiotics were stopped, but after the antibiotic regime was recommenced, symptom scores fell to levels reported prior to the suspension. Figure 1 includes a fourth data point from the most recent assessment, typically conducted by telephone and therefore not accompanied by urinalysis data.

**Table 1:** Results of repeated measures ANOVA comparing the variables collected at the three assessments

Variable	Df	F	Significance
Total symptoms score	2	33.228	0.000
Urgency symptoms	2	11.301	0.000
Stress urinary incontinence symptoms	2	4.282	0.014
Voiding symptoms	2	11.080	0.000
Pain symptoms	2	39.779	0.000
24-h frequency	2	1.008	0.366
24-h incontinence	2	3.795	0.023
Log_10_ wbc	2	3.713	0.026
Epithelial cell count	2	5.855	0.003

**Fig. 2:**
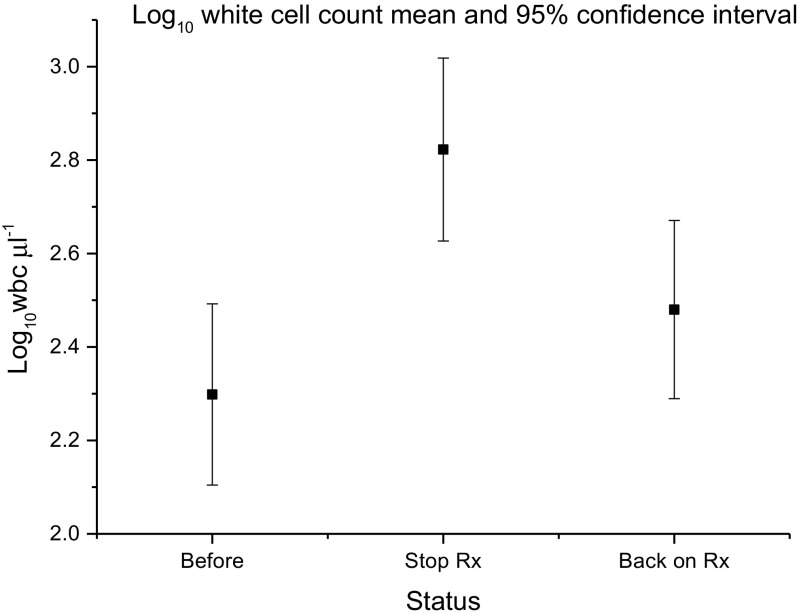
Mean pyuria (WBC) count on fresh urine microscopy before cessation, when off treatment and following reintroduction

Because of geographical dispersion and use of telephone review, urinalysis data were collected less often than symptom measures. Overall, urinalysis data were available for 132 patients (66%) before cessation, 130 patients after cessation (65%) and 122 (61%) after treatment reintroduction. Figure 2 plots the pyuria count before cessation, when off treatment and following reintroduction. There was an increase in urinary leucocyte expression after treatment cessation and a commensurate decrease after reinstating treatment (F = 3.7; df = 2; *p* = 0.026).

**Fig. 3:**
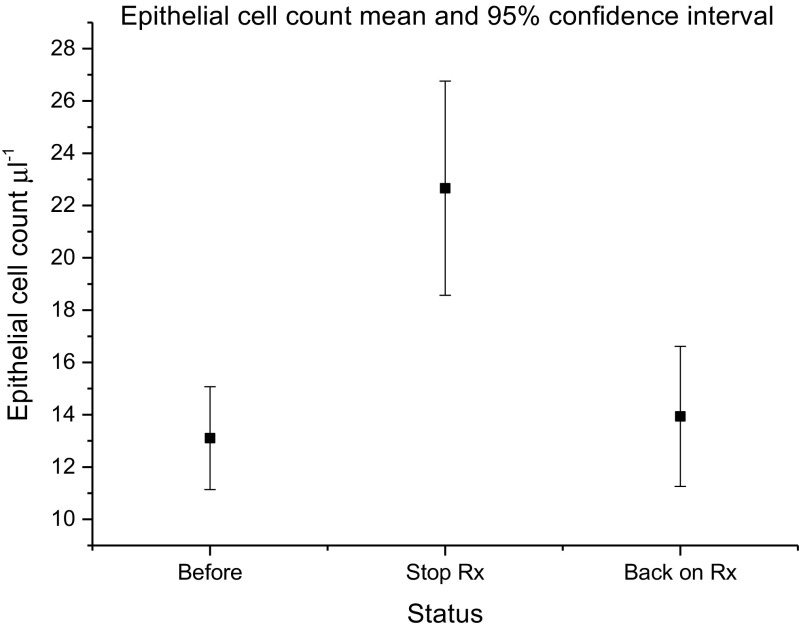
Mean epithelial cell count on fresh urine microscopy before cessation, when off treatment and following reintroduction

Figure 3 provides a similar analysis of the urothelial cell counts at different time points. Urothelial cell expression mirrored changes in symptom scores and urinary leucocyte numbers (F = 6.0; df = 2; *p* = 0.003). Twenty-four-hour frequency did not change over the series (F = 1; df = 2; *p* = 0.4) although 24-h incontinence worsened off treatment (F = 4.0; df = 2; p = 0.003). Menopausal status did not predict symptom scores, pyuria or epithelial cell counts.

Urine cultures were obtained at the first three time points, but only 20 (15%) were positive before treatment cessation, 24 (18%) after stopping and 25 (20%) after restarting treatment.

Eleven patients needed admission to hospital during the clinic closure and these were classified as SAEs in line with ICH GCP criteria [17]. Admission indications are summarised in Table 2. With one exception, these patients had not experienced any hospital admission for UTI-associated SAEs whilst receiving treatment from this service. The exception was one patient who previously required admission following interruption of the management regimen as part of our treatment protocol.

**Table 2:** Hospitalised patients with SAEs in association with treatment cessation

No. of patients (*n* = 11)	Description of SAEs and management
1	Paraplegia due to activation of autoimmune vasculitis secondary to urosepsis. Treated with cycles of IV methylprednisolone and cyclophosphamide; UTI remains difficult to manage
1	Admitted with urosepsis and CT evidence of right renal abscess. Required 6 weeks of IV antibiotic treatment
5	Admitted with acute UTI treated with a single-dose IV antibiotic treatment and 5 days of oral antibiotics after which UTI recurred
2	Reactivation of systemic lupus erythematous (SLE)
2	Worsening of multiple sclerosis (MS) symptoms and CISC for retention

## Discussion

These results support the hypothesis that treating painful LUTS with long-term antimicrobial courses is effective. These data support a mounting body of evidence [8, 9] that painful LUTS may have an infectious aetiology when dipstick and microbiological culture results do not indicate disease. The standard laboratory tests used to diagnose infection were positive only in a small proportion of the clinic’s symptomatic population (< 20%). This result is not surprising as we do not use MSU cultures to initiate treatment, and the insensitivity of urine culture is now well recognised [8].

An unplanned cessation of treatment in an unselected group of patients with chronic painful LUTS managed with protracted antimicrobial courses was coincident with a measurable deterioration in symptoms. The reintroduction of the original treatment regimen was associated with improvement. Only 24-h urinary frequency was unaffected. Arguing against a mere placebo effect or a by-product of the stress induced by the clinic suspension, the inflammatory biomarker pyuria mirrored the rise and fall of symptoms in these patients. Similarly, shed urothelial cell counts, which are known to reflect a host innate response to inflammation, moved in concert with the other variables, thus supporting a growing awareness of their ability to reflect the disease process [16, 18, 19].

A subset of the patients was hospitalised during the suspension period, and the underlying reasons for admission highlight worsening urinary infection, including pyelonephritis and urosepsis, in the face of antibiotic withdrawal. These events point to potentially significant consequences when patients receiving antibiotic therapy for presumed chronic infection have their treatment suddenly withdrawn without access to treatment reinstatement. It should be noted that all but one of these patients had remained free of hospital admissions whilst under the care of the service and receiving treatment.

To date, a limited number of RCTs support longer-term antibiotic treatment for painful LUTS caused by chronic infection [21–23]. The current data are not from an RCT, but they resulted from a random withdrawal of treatment followed by reinstating the same treatment. These circumstances provide credible observational data from a cross-over process. As well as corroborating the earlier RCTs, they are in agreement with our own previous observational data demonstrating improvements associated with treatment [9]. Our earlier paper also reported high relapse rates related to planned trials without treatment. These data suggest that presumed chronic infection may require much longer antibiotic courses than are recommended for other urological infections. The next step is a RCT using these data as proof of concept.

The principal limitation of this study is the lack of corroborative microbiological data to compliment the existing variables that we report. Our research programme and others have demonstrated the insensitivity of routine urine culture [7, 20–23], and treatment in the clinical service has not been contingent on routine culture evidence of UTI. In fact, our recent work shows that the MSU culture cannot distinguish this sort of patient from healthy controls, whereas a genomic analysis demonstrates clear differences (Sathiananthamoorthy et al., 2018, Journal of Clinical Microbiology JCM01452-18R1 In Press) [24]. Nonetheless, we recognise that the absence of MSU culture data will be contentious for many readers. More sensitive urinalysis methods have been reported in the literature, but their clinical application has yet to be evaluated [7, 25]*.*

Pyuria, however, is a well-established and independent biomarker of UTI and is not subject to the same bias as patient-reported symptoms. Once a staple of general clinical practice, fresh urine microscopy is an easy method, but it may be difficult to reintroduce this into ordinary healthcare. However, there are automated cell counters that are not expensive, which could be adapted to this role. We did not collect data on vaginal oestrogen use in post-menopausal women. Oestrogen replacement is not known to affect pyuria counts and we failed to see an effect during observational work.

Another limitation is that this study is not a clinical trial, though an RCT is being planned. However, the unique circumstances allowed us to generate data similar to that produced in cross-over studies, providing a rare opportunity to test our hypothesis.

Finally, only two thirds of patients generated urinalysis data, with the potential to generate selection bias. During normal clinic service, patients on telephone review had lower symptom scores than those attending in person for urinalysis (*t* = −3.2, *p* = 0.002, 95% CI of difference = −4.8 to −1.1), indicating more stable disease permitting remote surveillance. This was also the case after the prescribing restrictions were lifted and normal service resumed (*t* = −4.9, *p* < 0.001, 95% CI of difference = −5.9 to −2.5). By contrast, during the suspension there was no difference between symptom scores comparing those who attended for face-to-face review and urinalysis with those who were reviewed by telephone (*t* = −0.84, *p* = 0.4, 95% CI of difference = −2.4 to +1.0). Thus, it is less likely that the data were confounded by a selection bias.

The UK National Institute for Health and Care Excellence (NICE) has generated guidelines for the management of acute UTI, which limits antibiotic prescriptions to 14 days. The majority of patients in this study had failed to respond to standard antibiotic treatment and other recommended interventions for painful LUTS, prior to referral to this service, and a significant proportion of those referred for treatment came from other tertiary units. NICE has acknowledged a guideline deficit for patients affected by complicated UTI by publishing a “placeholder” statement, employed to identify a knowledge gap [10]. It is noteworthy that the treatment of acute UTI using recommended antibiotic prescribing guidelines is associated with a 25%–35% microbiological and symptomatic failure rate [26]. These data, and the results of our work, demonstrate the need for further research into the management of UTI and for existing guidelines on UTI to be urgently reviewed. It is our hope that clinicians will recognise the challenges that these patients pose in their own practices and that this will galvanise efforts to advance our knowledge in this neglected area of medicine.

While widespread concern about antimicrobial resistance (AMR) makes stewardship policies imperative, a one-size-fits-all approach may be equally unhelpful. Long-term antimicrobials should not be withheld when they are necessary (as is the case, for example, in tuberculosis patients). This is particularly true when patients are treated in a consultant-led clinical setting, subject to additional governance processes, careful monitoring and individualised therapy.

## References

[1] Barua JM, Arance I, Angulo JC, Riedl CR (2016). A systematic review and meta-analysis on the efficacy of intravesical therapy for bladder pain syndrome/interstitial cystitis. Int Urogynecol J.

[2] Dawson TE, Jamison J. Intravesical treatments for painful bladder syndrome/interstitial cystitis. The Cochrane Database of Systematic Reviews 2007;(4):CD006113. 10.1002/14651858. 10.1002/14651858.CD006113.pub2 PMC13389910 17943887

[3] Olson LE, Dyer JE, Haq A, Ockrim J, Greenwell TJ. A systematic review of the literature on cystodistension in bladder pain syndrome. Int Urogynecol J. 2017. 10.1007/s00192-017-3355-y. 10.1007/s00192-017-3355-y 28550461

[4] Basu M, Khullar V, Duckett J (2014). Urethral dilatation: is there any benefit over cystoscopy and distension? A randomized trial in women with overactive bladder symptoms. Neurourol Urodyn.

[5] Barber AE, Norton JP, Spivak AM, Mulvey MA (2013). Urinary tract infections: current and emerging management strategies. Clin Infect Dis.

[6] Smith AL, Brown J, Wyman JF, Berry A, Newman DK, Stapleton AE. Treatment and prevention of recurrent lower urinary tract infections in women: a rapid review with practice recommendations. J Urol. 2018. 10.1016/j.juro.2018.04.088. 10.1016/j.juro.2018.04.088 29940246

[7] Khasriya R, Sathiananthamoorthy S, Ismail S, Kelsey M, Wilson M, Rohn JL (2013). Spectrum of bacterial colonization associated with urothelial cells from patients with chronic lower urinary tract symptoms. J Clin Microbiol.

[8] Price TK, Hilt EE, Dune TJ, Mueller ER, Wolfe AJ, Brubaker L. Urine trouble: should we think differently about UTI? Int Urogynecol J. 2017. 10.1007/s00192-017-3528-8. 10.1007/s00192-017-3528-8 29279968

[9] Swamy S, Barcella W, De Iorio M, Gill K, Khasriya R, Kupelian AS, et al. Recalcitrant chronic bladder pain and recurrent cystitis but negative urinalysis: what should we do? Int Urogynecol J. 2018. 10.1007/s00192-018-3569-7. 10.1007/s00192-018-3569-7 PMC6004281 29556674

[10] NICE. Urinary tract infection (lower) - women. NICE guidelines [Internet]. 2014. Available from: http://cks.nice.org.uk/urinary-tract-infection-lower-women#!topicsummary.

[11] Kupelian AS, Horsley H, Khasriya R, Amussah RT, Badiani R, Courtney AM (2013). Discrediting microscopic pyuria and leucocyte esterase as diagnostic surrogates for infection in patients with lower urinary tract symptoms: results from a clinical and laboratory evaluation. BJU Int.

[12] Gill K, Jeyakumar A, Brenton T, Malone-Lee J (2010). A urine test for OAB. Int Urogynecol J.

[13] Holmberg L, Boman G, Bottiger LE, Eriksson B, Spross R, Wessling A (1980). Adverse reactions to nitrofurantoin. Analysis of 921 reports. Am J Med.

[14] 2015 NEsEPdM. Clostridium difficile infection: risk with broad-spectrum antibiotics. Published date: March 2015.

[15] Khasriya R, Barcella W, De Iorio M, Swamy S, Gill K, Kupelian A, et al. Lower urinary tract symptoms that predict microscopic pyuria. Int Urogynecol J. 2017. 10.1007/s00192-017-3472-7. 10.1007/s00192-017-3472-7 PMC6004270 28971220

[16] Horsley H, Malone-Lee J, Holland D, Tuz M, Hibbert A, Kelsey M, et al. Enterococcus faecalis subverts and invades the host urothelium in patients with chronic urinary tract infection. PLoS One. 2013;8(12). 10.1371/journal.pone.0083637 PMC3868479 24363814

[17] Englev E, Petersen KP (2003). ICH-GCP guideline: quality assurance of clinical trials. Status and perspectives. Ugeskr Laeger.

[18] Horsley H, Dharmasena D, Malone-Lee J, Rohn JL (2018). A urine-dependent human urothelial organoid offers a potential alternative to rodent models of infection. Sci Rep.

[19] Choi HW, Bowen SE, Miao Y, Chan CY, Miao EA, Abrink M (2016). Loss of bladder epithelium induced by cytolytic mast cell granules. Immunity.

[20] Gill K, Kang R, Sathiananthamoorthy S, Khasriya R, Malone-Lee J. A blinded observational cohort study of the microbiological ecology associated with pyuria and overactive bladder symptoms. Int Urogynecol J. 2018. 10.1007/s00192-018-3558-x. 10.1007/s00192-018-3558-x PMC6154027 29455238

[21] Pearce MM, Hilt EE, Rosenfeld AB, Zilliox MJ, Thomas-White K, Fok C (2014). The female urinary microbiome: a comparison of women with and without urgency urinary incontinence. mBio.

[22] Wolfe AJ, Toh E, Shibata N, Rong R, Kenton K, Fitzgerald M (2012). Evidence of uncultivated bacteria in the adult female bladder. J Clin Microbiol.

[23] Fok CS, Gao X, Lin H, Thomas-White KJ, Mueller ER, Wolfe AJ, et al. Urinary symptoms are associated with certain urinary microbes in urogynecologic surgical patients. Int Urogynecol J. 2018. 10.1007/s00192-018-3732-1. 10.1007/s00192-018-3732-1 PMC6830733 30116843

[24] Sathiananthamoorthy S. PhD the microbiology of chronic lower urinary tract symptoms. UCL: UCL; 2016.

[25] Hilt EE, McKinley K, Pearce MM, Rosenfeld AB, Zilliox MJ, Mueller ER (2014). Urine is not sterile: use of enhanced urine culture techniques to detect resident bacterial flora in the adult female bladder. J Clin Microbiol.

[26] Milo G, Katchman EA, Paul M, Christiaens T, Baerheim A, Leibovici L. Duration of antibacterial treatment for uncomplicated urinary tract infection in women. Cochrane Database Syst Rev 2005;(2):CD004682. 10.1002/14651858.CD004682.pub2 PMC12700497 15846726

